# Health effects of wildfire PM_2.5_ in Latin American cities: A rapid systematic review and comparative synthesis

**DOI:** 10.7705/biomedica.8068

**Published:** 2025-11-27

**Authors:** Jeadran Malagón-Rojas, Kai Chen

**Affiliations:** 1 Observatorio Nacional de Salud, Instituto Nacional de Salud, Bogotá, D. C., Colombia Instituto Nacional de Salud Instituto Nacional de Salud Bogotá, D. C. Colombia; 2 Yale Center on Climate Change and Health, Yale Institute for Global Health, New Haven, CT, USA Yale Institute for Global Health Yale Institute for Global Health New Haven CT USA

**Keywords:** wildfires, climate change, particulate matter, mortality, morbidity, Latin America., incendios forestales, cambio climático, material particulado, mortalidad, morbilidad, Latinoamérica

## Abstract

**Introduction.:**

Wildfire activity is intensifying in Latin America due to climate and land-use changes, but the health impacts of wildfire-derived PM_2.5_ in urban areas remain poorly quantified and recognized.

**Objective.:**

To assess the evidence on wildfire-related PM_2.5_ and its association with mortality and morbidity in Latin American cities.

**Materials and methods.:**

We conducted a rapid systematic review and meta-analysis following PRISMA guidelines, using data from PubMed, Scopus, and Bireme. One reviewer independently screened 163 articles and extracted data from 14 eligible studies. A risk of bias assessment was conducted using the Newcastle-Ottawa Scale.

**Results.:**

Most studies were conducted in Brazil (n = 12) and used time-series or modelling designs to estimate health risks. Wildfire-specific PM_2.5_ exposure was associated with allcause, cardiovascular, and respiratory mortality. Reported effect estimates ranged from 1.7 to 7.7% increases in risk per 10 μg/m^3^ of exposure. Other studies assessed preterm birth, COVID-19 outcomes, and site-specific cancers. While two studies provided harmonized RR estimates for all-cause mortality, high heterogeneity and methodological differences prevented formal meta-analysis.

**Conclusion.:**

Wildfire smoke contributes measurably to premature mortality in Latin America, but current evidence is unevenly distributed across regions, time periods, and population subgroups. Studies rarely capture the disproportionate risks faced by indigenous and rural communities or the intraurban disparities linked to poverty and geography. Future research should focus on the health burden of morbidity linked to wildfire PM_2.5_.

## Resumen

**Introducción.:**

La actividad de los incendios forestales se está intensificando en Latinoamérica debido al cambio climático y al uso del suelo, aunque los impactos en la salud del material particulado fino (PM_2.5_) derivado de estos incendios en las áreas urbanas siguen estando poco cuantificados y reconocidos.

**Objetivo.:**

Evaluar la evidencia sobre el PM_2.5_ relacionado con los incendios forestales y su asociación con la mortalidad y morbilidad en las ciudades latinoamericanas.

**Materiales y métodos.:**

Se realizó una revisión sistemática rápida y un metaanálisis siguiendo las directrices PRISMA, utilizando datos de PubMed, Scopus y Bireme. Un revisor examinó de forma independiente 163 artículos y extrajo datos de 14 estudios que se elegieron. Se llevó a cabo una evaluación del riesgo de sesgo utilizando la escala de Newcastle-Ottawa.

**Resultados.:**

La mayoría de los estudios se llevaron a cabo en Brasil (n = 12) y emplearon diseños de series temporales o modelos para estimar los riesgos para la salud. La exposición a PM_2.5_ -específico de los incendios- se asoció con mortalidad por todas las causas, cardiovascular y respiratoria. Las estimaciones del efecto reportadas oscilaron en aumentos del 1,7 al 7,7% en el riesgo por cada incremento de 10 μg/m^3^ de exposición. Otros estudios evaluaron partos prematuros, desenlaces por COVID-19 y cánceres específicos por sitio. Aunque dos estudios proporcionaron estimaciones armonizadas de riesgo relativo para la mortalidad por todas las causas, la alta heterogeneidad y las diferencias metodológicas impidieron realizar un metaanálisis formal.

**Conclusiones.:**

El humo de los incendios forestales contribuye de manera significativa a la mortalidad prematura en Latinoamérica, pero la evidencia actual está distribuida de forma desigual entre regiones, períodos de tiempo y subgrupos poblacionales. Rara vez se identifican los riesgos desproporcionados que enfrentan las comunidades indígenas o rurales, las disparidades intraurbanas vinculadas a la pobreza y la geografía. Las investigaciones futuras deberían centrarse en la carga de morbilidad asociada con el PM_2.5_ de los incendios forestales.

In recent years, “wildfires” have become a global buzzword frequently invoked in climate change discourse, media narratives, and international policy frameworks. While this framing has helped to draw attention to the growing frequency and severity of fire events worldwide, it often obscures the complex, context-specific realities of fire in the Global South. In Latin America, for instance, many fires are not spontaneous outcomes of climate extremes, but rather deliberate, seasonal, and culturally embedded practices tied to land management, agricultural cycles, and territorial dynamics ^1^^-^^3^, conflating all fire events under the umbrella of climate-driven disaster risks flattening this diversity and misrepresenting the true nature of fire exposure in the region.

Latin America is a global hotspot for biomass burning, with recurrent fire activity in the Amazon, Pantanal, and Cerrado biomes ^4^^-^^7^. Yet, the health literature in the region has historically prioritised chronic urban air pollution over episodic but severe fire-related exposures ^8^. Cities such as Manaus, Bogotá, and São Paulo are periodically affected by transboundary smoke, but the epidemiological evidence on associated health outcomes remains fragmented and methodologically heterogeneous ^9^.

While global burden assessments have estimated that fire-derived PM_2.5_ contributes significantly to premature mortality, some studies have applied fire-specific exposure attribution in Latin American urban contexts ^10^. While most studies to date have focused on mortality outcomes, wildfire- related PM_2.5_ exposure is also associated with a wide range of sublethal health effects, including respiratory exacerbations, cardiovascular stress, and adverse pregnancy outcomes, effects that remain largely undocumented in Latin American cities. Importantly, the spatial and temporal dynamics of fire exposure in Latin America differ markedly from those in high-income countries. In the United States, Canada, and Australia, wildfires are now a dominant source of PM_2.5_ in many urban areas ^11^^,^^12^. In contrast, Latin American cities may experience more sporadic fire influence, with substantial variation across subregions. For instance, cities in the Andean highlands and Central America are typically insulated from nearby fire activity by geographic distance from typical burn regions, except during exceptional events that capture public and political attention. A recent example occurred in Bogotá in 2024, when smoke from fires in the Amazon and eastern plains triggered an air quality crisis and emergency health alerts ^13^^,^^14^.

In many parts of Latin America, particularly in the Andes highlands and Amazon basin, fire is not merely a natural hazard but a culturally embedded land management tool. Agricultural burning (used to clear land, stimulate pasture regrowth, and manage pests) remains widespread, especially in agropastoral systems in highland Perú and Bolivia, but also in Venezuela, Colombia and Brazil ^3^^,^^15^. These practices are often seasonal, concentrated in the dry months (July to November), and closely tied to traditional knowledge and land tenure systems. However, shifts toward individual land ownership, erosion of communal governance, and climate variability have increased the risk of uncontrolled wildfires. Despite regulatory bans, enforcement remains limited, and fire continues to be used during specific windows of the agricultural calendar.

These dynamics differ from those in the Global North, where wildfires are often framed as climate-driven disasters. In Latin America, the question is not only how much PM_2.5_ is produced by fires, but when, where, and why these fires occur, and how their health impacts compared to other pollution sources. However, such evidence remains the exception rather than the rule across the region. In countries like Panamá, for example, low-cost air quality sensors have only recently begun to reveal the extent of pollution in high-traffic urban areas, with limited integration of fire-related sources. This uneven landscape of data availability and methodological capacity further justifies the need for a regionally focused synthesis of fire-related health impacts in Latin American cities.

This review seeks to consolidate and critically appraise the available evidence on the short-term mortality impacts of wildfire-related PM_2.5_ in Latin American cities. While previous global studies have included the reg^.^ ion in broader analyses, few have focused specifically on Latin America or disaggregated results by country or subregion. Our aim is not to provide a comprehensive meta-analysis across all outcomes, but rather to synthesize the best available data to inform public health planning in a region where fire activity is intensifying and health system preparedness remains uneven.

We followed a pre-registered protocol aligned with PRISMA 2020 guidelines. Screening and data extraction were conducted by a trained reviewer, with a validation step to ensure retrieval of key reference studies. Where effect estimates were sufficiently harmonised, we performed quantitative metaanalysis; otherwise, we applied structured narrative synthesis.

Although global discourse often frames wildfires as climate-driven disasters, in Latin America, many fires are linked to land-use practices, agricultural cycles, and deforestation. This complexity challenges standard exposure assessments. Most epidemiological studies in the region aggregate health outcomes without spatial disaggregation, obscuring community-specific risks. This review aims to clarify the short-term health effects of wildfire-related PM_2.5_ in Latin American urban contexts and highlight critical evidence gaps.

In this context, this review aims to inform both scientific and policy communities about the acute mortality and morbidity risks associated with fire-related air pollution in Latin American urban environments.

## Materials and methods

This rapid systematic review was conducted according to a pre-registered protocol on the Open Science Framework (osf.io/6n3uf) ^16^ and adheres to PRISMA 2020 guidance ^17^. The aim was to synthesize available evidence on the impact of wildfire-related fine particulate matter (PM_2.5_) on mortality and morbidity outcomes in urban areas of Latin America, considering also how these effects may be modified by socioeconomic and health service disparities.

The review followed a structured multi-stage process, beginning with the development and registration of a protocol that defined the review questions, eligibility criteria, and synthesis plan. We searched three databases: PubMed, Scopus, and Bireme, covering literature published from 2010 to 2025. Boolean search strategies were tailored for each database, and included controlled vocabulary and free-text terms relating to wildfires, PM_2.5_ exposure, mortality, and Latin American urban settings. Complete search strings for each database are available in the Open Science Framework repository.

Because the review was carried out by a single trained reviewer, no dual screening or extraction was performed. To enhance rigour and transparency despite this limitation, we implemented a search validation procedure. Specifically, we identified a small set of key studies known to be highly relevant, based on prior scoping and expert knowledge. The search algorithms were iteratively adjusted until these key references, such as Chen *et al*. ^10^, Ye *et al*. ^18^, and Requia *et al*. ^19^, were successfully retrieved. This process helped ensure the sensitivity and adequacy of the search strategy.

All retrieved records were imported into Rayyan, a web-based tool designed to manage screening workflows in systematic reviews ^20^. After automatic deduplication using Rayyan’s web platform, the reviewer conducted title screening, followed by combined title and abstract screening, and ultimately full-text assessment. Articles were excluded at each stage based on predefined criteria specified in the protocol, including geographic scope (non-Latin American), lack of wildfire attribution, absence of mortality or hospitalisation outcomes, or insufficient methodological detail.

Blinding of bibliographic metadata (authors, journal names, institutions) was not implemented, since the review was carried out by one person, and masking would not meaningfully reduce bias in a single-reviewer context. Instead, the reviewer followed a structured decision-making protocol, with exclusion criteria applied consistently and documented at each stage.

**Figure 1:**
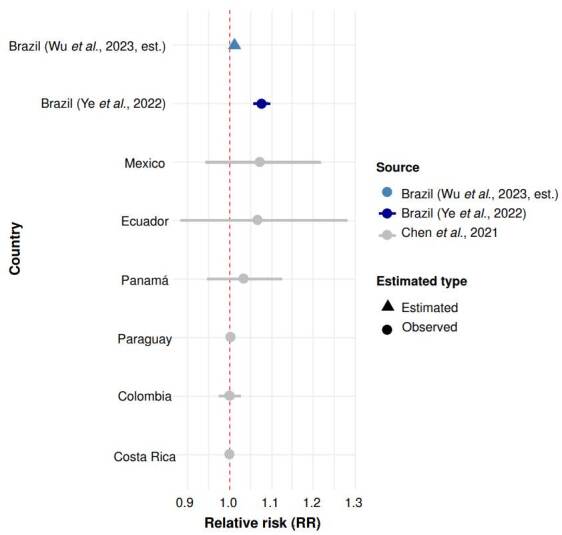
Respiratory mortality associated with wildfire PM_2.5_ in Latin America. Relative risks (RR) and 95% confidence intervals (CI) for respiratory mortality per 10 μg/m^3^ increase in wildfire- related PM_2.5_ exposure. Estimates are shown for six Latin American countries and an estimate for Brazil from Ye *et al*. ^18^. An additional RR for Brazil was back-calculated from the attributable fraction (AF = 1.25%) reported by Wu *et al*. ^25^ and is presented without a confidence interval. The red dashed line indicates the null value (RR = 1.0). The estimate from Ye *et al*. ^18^ is highlighted in dark blue; the back-calculated estimate from Wu *et al*. ^25^ is shown in light blue and marked as estimated.

For each included article, data were extracted using a structured spreadsheet designed to capture the following variables: study identification (authors, year, title), country and city, type of fire, PM_2.5_ exposure measurement method, study design, population group, outcome(s) (all-cause, cardiovascular, respiratory mortality), covariates included, effect estimates (*i.e*., RR, OR, β with 95% CI), author-stated limitations. Exposure metrics were standardised to per 10 μg/m^3^ PM2 5 when possible. Extraction was conducted using full texts, figures, and supplementary materials. Ambiguities or missing data were flagged, and studies for which critical variables could not be retrieved were noted in the extraction file. For the study by Wu *et al*. ^25^, which reported an attributable fraction of 1.25% for respiratory mortality associated with wildfire-related PM_2.5_ exposure in Brazil, the relative risk (RR) was not directly provided. As the corresponding author did not respond to repeated contact attempts, we back-calculated the RR using the inverse of the attributable fraction formula ^21^. Using the reported attributable fraction (0.0125), we estimated an RR of approximately 1.0127. This value was included in the analysis and visualized in figure 1 as an estimated point without a confidence interval, solely for illustrative purposes.

We initially planned a meta-analysis; however, given the small number of harmonised effect estimates and substantial heterogeneity (l^2^ > 85%), we opted to present forest plots descriptively, without pooling. Details of excluded studies and reasons for exclusion are provided in the PRISMA diagram.

The Newcastle-Ottawa Scale was applied to assess methodological quality ^22^. This version rated studies on three domains: (1) population and exposure selection, (2) control for confounders, and (3) outcome assessment. Each study was scored out of 9 and classified as low (0-4), moderate (5-6), or high (≥ 7) quality. These scores were documented and used in sensitivity analyses. Full scoring details are provided in supplementary table 1.

**Table 1:** Characteristics of the studies included in the review.

Study (author, year)	Country	Design	Outcome	Effect estimate	Exposure (model)	Reason for exclusion
Ye *et al*. ^18^	Brazil	Time-series	All-cause, respiratory, cardiovascular mortality	RR 1.031 (all-cause), 1.026 (cardio), 1.077 (resp) per 10 pg/m^3^	GEOS-Chem + calibration	Included
Chen *et al*. ^10^	Multi-country (incl. LATAM)	Time-series	All-cause, respiratory, cardiovascular mortality	RR 1.019 (all-cause), 1.017 (cardio), 1.019 (resp) per 10 pg/m^3^	GEOS-Chem	-
Wu *et al*. ^25^	Brazil	Modelling	All-cause, respiratory mortality	AF 1.25% (all-cause)	GEOS-Chem + ML	Reported AF, RR was calculated
Gao *et al*. ^32^	Brazil	Quasiexperimental	Cardiovascular mortality, hospitalisation	RR 1.031 (IHD), 1.020 (stroke)	GEOS-Chem + RF	Individual cardiovascular causes (IHD, stroke), not aggregated for CVC, non-standard exposure binning
Ballesteros-González *et al*. ^37^	Colombia	Modelling	Respiratory, all-cause mortality	88 excess deaths	WRF-Chem	Absolute excess deaths, not RR
Cobelo *et al*. ^35^	Brazil	Ecological	All-cause mortality	Excess mortality (penalty metric)	CAMS	Not RR or OR
Requia *et al*. ^19^	Brazil	Case-crossover	Preterm birth	OR 1.05-1.41	CAMS	OR, reproductive outcome
Yu *et al*. ^26^	Brazil	DiD (ecological)	Site-specific cancer mortality	RR 1.02 per 1 µg/m3	GEOS-Chem	RR for cancer outcome
Gonçalves *et al*. ^33^	Brazil	Ecological	COVID-19 incidence & mortality	RR 1.8 (incidence), 1.5 (mortality)	Satellite-derived	COVID-19 outcome, short time frame
Lorenz *et al*. ^34^	Brazil	Ecological	COVID-19 hospitalisations	+23% increase	Satellite-derived	Hospitalisations only; ecological inference
Nunes *et al*. ^2^	Brazil	Ecological	Cardiovascular mortality	Not reported (p values only)	CATT-BRAMS	No RR or quantitative estimate
Nawaz and Henze ^28^	Brazil	Modelling	Cause-specific mortality (IHD, stroke, etc.)	4,407 premature deaths	GEOS-Chem adjoint	Absolute number of deaths
Butt *et al*. ^29^	Brazil	Modelling	Premature mortality	3,400 deaths in 2019	WRF-Chem	Absolute number of deaths

Exposure estimation methods:

GEOS-Chem: atmospheric chemical transport model; Satellite-derived: based on AOD (MODIS) combined with regression or interpolation; WRF: weather and air quality model; CAMS: reanalysis-based atmospheric data; ML: machine learning-based PM2.5 prediction

A narrative synthesis was performed, grouping studies by outcome type, study design, and regional context. Tables and visual summaries were created to compare study characteristics, highlight methodological differences, and synthesise findings thematically.

All data extraction tables, Newcastle-Ottawa Scale assessments, and analysis scripts were archived and made publicly available through the Open Science Framework repository, in compliance with FAIR (Findable, Accessible, Interoperable, and Reusable) data principles ^23^ and funding requirements of the Global Health Emerging Scholars Program under award number D43TW010540.

Although fire events in Latin America include both wildfires and prescribed or agricultural burns, most studies included in this review did not explicitly distinguish between fire types (*i.e*., naturally occurring or human-caused). Emission inventories used in exposure models, such as the Fire Inventory from NCAR, the Global Fire Emissions Database, and the Global Fire Assimilation System, often aggregate fire emissions without disaggregation by ignition source. In regional studies, the Brazilian Biomass Burning Emission Model provides detailed emissions estimates specific to Brazil. As a result, estimated associations with PM_2.5_ exposure may reflect a combination of fire types, potentially conflating distinct socio-environmental processes.

## Results

**Figure 2:**
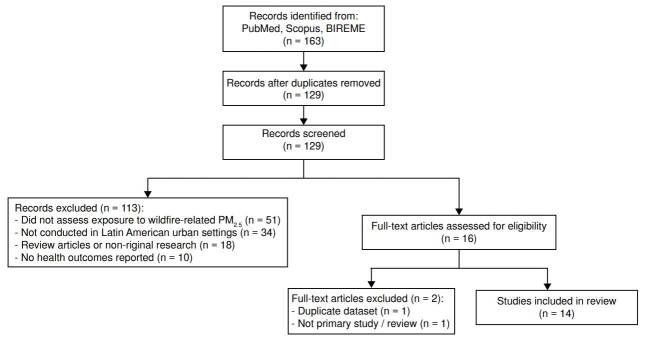
PRIMSA flow diagram

The 14 included studies applied a range of observational designs. Three used time-series analyses, commonly considered a subtype of ecological studies, though analysed separately here, due to their temporal resolution and daily exposure modelling. Four studies applied ecological designs, including one purely cross-sectional comparison ^2^^,^^33^^-^^35^, two retrospective time-series with aggregated annual data ^10^^,^^18^, and one spatial ecological analysis using geographic aggregates ^26^. Two studies employed quasi-experimental frameworks (*i.e*., difference-in-differences), one used a case-crossover design, and four relied on modelling-based impact assessments. Geographically, the studies ranged from single-city evaluations (*e.g*., Bogotá ^24^, Manaus ^29^ to analyses encompassing all 5,565 municipalities in Brazil (table 1). The geographic distribution of studies revealed a substantial focus on the Brazilian Amazon ^2^^,^^25^^-^^35^, and South America ^10^ (figure 2).

Wildfire-related PM_2.5_ exposure was estimated using advanced atmospheric models in most studies, primarily GEOS-Chem and WRF- Chem, often calibrated with ground-based monitors and machine learning techniques. Exposure periods ranged from a single burn season to 16 years. In 11 studies, PM_2.5_ was explicitly attributed to vegetation fires, while two studies used broader land-use categories without isolating wildfire emissions. A detailed summary of epidemiological designs, exposure assessment methods, and Newcastle-Ottawa Scale ratings for all included studies is provided in supplementary table 1.

### All-cause mortality

Five studies reported associations between wildfire-related PM_2.5_ and allcause mortality. Ye *et al*. ^18^ estimated a 3.1% increase in mortality per 10 μg/m^3^ (RR = 1.031; 95% CI: 1.024 - 1.039) in Brazil. Chen *et al*. ^10^, using a global dataset, reported a 1.9% increase (RR = 1.019; 95% CI: 1.016 - 1.022) for South America. These estimates are visualized in figure 2, which presents a lollipop plot comparing relative risks across regions. The figure highlights the higher effect size observed in Brazil compared to the regional average, although both estimates suggest a positive association.

Two studies ^10^^,^^21^ provided harmonized relative risk estimates for allcause mortality using similar time-series designs and comparable exposure metrics. While we initially considered pooling these results, we ultimately opted against a meta-analysis due to high heterogeneity (l^2^ = 88.3%) and limited statistical power. Instead, we present them descriptively to inform regional interpretation. One study ^25^ reported an attributable fraction, from which we derived a relative risk using epidemiological transformation.

**Figure 3:**
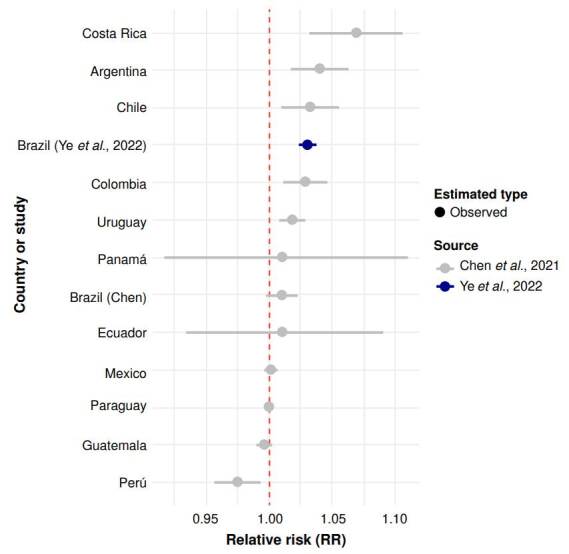
Lollipop plot of RR for all-cause mortality linked to wildfire PM_2.5_ in Latin America. Lollipop plot displaying relative risk (RR) and 95% confidence intervals (CI) for all-cause mortality per 10 µg/mP increase in wildfire-related PM_25_ exposure. Country-specific estimates are from Chen *et al*. (10), with an estimate for Brazil from Ye *et al*. _^(18)^_ . The red dashed line indicates the null value (RR = 1.0).

Instead, we present a comparative visualization of these estimates across Latin American countries (figure 3), highlighting regional variability in estimated risk. Brazil, Costa Rica, Chile, Colombia, and Uruguay had among the highest point estimates of risk, while countries like Guatemala, Mexico, Paraguay and Perú showed null effects (figure 3). These differences may reflect disparities in fire regimes, exposure duration, and urban vulnerability profiles.

### Cause-specific mortality

**Figure 4:**
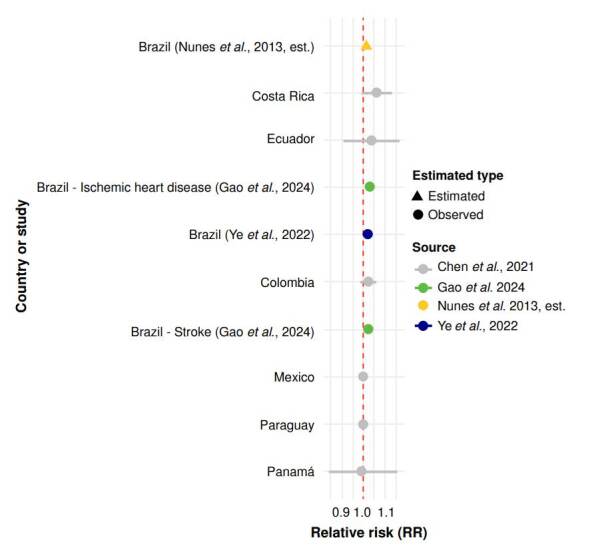
Cardiovascular mortality associated with wildfire PM_2.5_ in Latin America. Relative risks (RR) and 95% confidence intervals (CI) for cardiovascular mortality per 10 μg/m^3^ increase in wildfire-related PM_2.5_ exposure. Country-specific estimates are from Chen *et al*. _^(10)^_ , with an estimate for Brazil from Ye *et al*. _^(18)^_ . Additional cause-specific estimates for ischemic heart disease and stroke are from Gao *et al*. _^(32)^_ . An estimated RR from Nunes *et al*. _^(2)^_ is included without a confidence interval, as no RR was reported. The red dashed line indicates the null value (RR = 1.0).

For cardiovascular mortality, Ye *et al*. ^18^ and Chen *et al*. ^10^ reported increases of 2.6% and 1.7%, respectively. Gao *et al*. ^32^ found a 2.2% increase in total cardiovascular mortality, with stronger associations for ischaemic heart disease (RR = 1.031; 95% CI: 1.014 - 1.048) and stroke (RR = 1.020; 95% CI: 1.002 - 1.038). Nunes *et al*. ^2^ observed elevated mortality from myocardial infarction and cerebrovascular disease in elderly populations in the Brazilian Amazon, although no risk ratios were reported (figure 4).

Respiratory mortality yielded the largest effect sizes. Ye *et al*. ^18^ reported a 7.7% increase (RR = 1.077; 95% CI: 1.059 - 1.095), while Chen *et al*. ^10^ found a 1.9% increase (RR = 1.019; 95% CI: 1.017 - 1.022) (figure 1).

### Cancer mortality

One study ^26^ examined the association between wildfire-related PM_2.5_ and site-specific cancer mortality in Brazil. Using a difference-in-differences design with nationwide data from 2010 to 2016, the authors reported an overall relative risk of 1.02 (95% CI: 1.01 - 1.03) per 1 μg/m^3^ increase in PM_2.5_. Stronger associations were observed for specific cancer types, including prostate cancer (RR = 1.07; 95% CI: 1.03 - 1.11), testicular cancer (RR =1.09; 95% CI: 1.01 - 1.18), and colorectal cancer (RR = 1.04; 95% CI: 1.01 - 1.08). The study found consistent positive associations across multiple cancer sites, suggesting a possible carcinogenic role of biomass-derived PM_2.5._

### Other health outcomes

Hospital admissions were analysed in two studies. Ye *et al*. ^18^ reported a 1.65% increase in all-cause admissions, a 5.09% increase in respiratory hospitalisations, and a 1.10% increase in cardiovascular admissions per 10 μg/m^3^. Gao *et al*. ^32^ inferred elevated healthcare burden from excess mortality and exposure levels.

Preterm birth was assessed in one study ^19^, which found odds ratios ranging from 1.05 to 1.41 depending on region and trimester, with the highest risks observed in Brazil’s southeast, where more densely populated areas are located, such as São Paulo and Rio de Janeiro (OR = 1.14; 95% CI: 1.31 - 1.51), compared to the northern region (OR = 1.05; CI 95%: 1.01 - 1.09), suggesting a distinct geographical gradient.

Two studies examined COVID-19 outcomes in the Brazilian Amazon region. Gonçalves *et al*. ^33^ reported a 1.8-fold increase in COVID-19 incidence and a 1.5-fold increase in COVID-19 mortality per 10 μg/m^3^ increase in PM_25_. Lorenz *et al*. ^34^ documented a 23% increase in hospitalisations in the Pantanal region of Mato Grosso do Sul (Brazil), with effect modification by Gini coefficient inequality and meteorological conditions.

### Premature mortality burden

Two modelling studies -Butt *et al*. ^29^ and Nawaz and Henze ^28^- estimated premature deaths attributable to wildfire-related PM_2.5_ during the 2019 Amazon fire season. Butt *et al*. ^29^ estimated 3,400 deaths (95% CI: 3,300 - 3,550), while Nawaz and Henze ^28^ reported 4,966 deaths (95% CI: 2,426 - 8,380).

## Discussion

This systematic review highlights that short-term exposure to wildfire- related PM_2.5_ contributes to elevated mortality risks in Latin America. While several studies reported increased all-cause, cardiovascular, and respiratory mortality, we found substantial variation in exposure metrics and outcome definitions. Only two studies ^10^^,^^21^ provided harmonized estimates suitable for comparison, and even among these, methodological differences and statistical heterogeneity precluded formal meta-analysis. In one case, RR was derived from attributable fraction data due to lack of direct reporting.

Twelve of the fourteen studies included were conducted in Brazil, particularly in the Amazon basin. While this reflects both the ecological urgency and scientific capacity in the Amazon region, it limits the generalizability of findings across Latin America. Notably, data from Central America and the Andean region were limited in the evidence base. This imbalance further supports the need for targeted investment in underrepresented countries, especially in cities in expansion, and distinct from Buenos Aires, México City, Bogotá, São Paulo, Santiago, and Lima, where wildfire smoke may interact with complex urban pollution profiles.

Previous studies have established the health risks of biomass burning emissions, particularly in tropical regions, but few have focused on Latin American urban centres where transboundary smoke events are recurrent ^36^^-^^38^. This review confirms a growing body of evidence linking wildfire- derived PM_2.5_ to adverse health outcomes in Latin America, particularly in Brazil. The associations observed -for all-cause, cardiovascular, and respiratory mortality- are directionally consistent with studies from high- income countries, though modest in magnitude. Yet, beneath this apparent consistency lies a more complex narrative: despite the intensification of fires across the region, few urban centres in Latin America have been meaningfully studied. Most data come from national-scale modelling, rather than direct epidemiological surveillance in exposed populations. This gap raises questions not only about the magnitude of the effect, but about where and when fire-PM_2.5_ truly constitutes a public health concern in Latin America.

Wildfires are increasingly framed as a global environmental health threat. But in Latin America, they may not always be the dominant source of particulate pollution in urban settings. An important insight emerging from this review is the spatial disjunction between the origin of wildfire emissions and the populations in which health outcomes are measured. Most studies have focused on large urban centres -such as São Paulo, Rio de Janeiro, and Bogotá- or aggregated outcomes across thousands of municipalities, predominantly in Brazil. However, fire emission inventories (e.g., QFED, FINN) and exposure models (GEOS-Chem, WRF-Chem) have consistently identified the Amazon and other remote, rural regions as the primary sources of wildfire-related PM_2.5_. This suggests that while the smoke is transported over long distances to urban areas, the fires themselves typically occur far from the populations studied. Few studies addressed this spatial dynamic explicitly, and none examined differential exposure or risk across rural versus urban settings. The urban-centric focus of current epidemiological evidence may therefore underrepresent the burden in peri-urban or rural populations, particularly among indigenous and traditional communities who often inhabit fire-prone regions but remain poorly captured by national surveillance systems.

Moreover, the limited availability of granular outcome data constrains our understanding of who is most at risk. Several studies used aggregated, all-age, all-cause mortality as their primary endpoint, thereby masking heterogeneity across age groups, comorbidities, or socioeconomic strata. While some analyses stratified by age or sex to examine differential effects, few explored effect modifications by poverty, access to care, or geographic marginalisation. Critically, morbidity outcomes were underrepresented. Hospital admissions were assessed in only two studies, and none of the included studies evaluated subclinical respiratory symptoms, disability, or long-term disease progression attributable to wildfire smoke.

One reason for this gap may lie in structural limitations in health surveillance systems across the region. For instance, in countries like Colombia, morbidity datasets -such as those related to respiratory infections- are linked to the health care provider where the case was reported, rather than to the patient’s place of residence. This disconnection limits the ability to conduct ecological or spatial analyses of disease burden. while surveillance systems such as the severe acute respiratory infection network have recently incorporated geographic identifiers like urban planning zones, this information remains underutilised in environmental epidemiology. Without spatially resolved morbidity data, estimating the full health burden of wildfire pollution -especially among vulnerable urban populations- remains elusive.

Importantly, most studies could not be pooled due to heterogeneity in exposure metrics, outcome definitions, and statistical models. For instance, studies using odds ratios for binary outcomes, or those estimating population attributable fractions, could not be meaningfully integrated with time-series relative risks. We made a deliberate choice to disaggregate data from the multicountry study by Chen *et al*. (10), including only Latin American countrylevel estimates where available, rather than the global estimate. This allowed us to contextualise effects in the region but introduced further variability between studies. The l^2^ of 95% observed in our all-cause mortality model reflects this heterogeneity.

### Knowledge gaps and future research directions

Despite the growing number of studies addressing wildfire-related PM_2.5_ in Latin America, our review reveals notable blind spots. First, most studies aggregate outcomes at national or regional levels, often using designs and models that obscure intra-urban and inter-group disparities. This is problematic because air pollution exposure is not spatially homogeneous, even within the same city. For instance, in Bogotá (Colombia), school closures during wildfire smoke episodes have been concentrated in lower- income districts near forested hillside areas ^39^^,^^40^. Similarly, exposure among young children and older adults is disproportionately associated with elevated health risks, yet most studies present population-averaged effects that obscure age-specific susceptibilities. These patterns suggest that both environmental exposure and health vulnerability follow socio-spatial gradients that merit finer-resolution analysis.

Similarly, age-stratified or risk-based analyses are inconsistently applied. While some studies disaggregate outcomes by age or sex, few assess specific vulnerabilities among children under five or older adults. This dilutes risk estimates and undermines the precision needed for targeted public health interventions.

Importantly, the current evidence base privileges mortality over morbidity, overlooking more frequent and potentially more sensitive indicators of health burden. The absence of studies on asthma exacerbations, hospital admissions, or outpatient visits likely reflects structural limitations in health information systems, particularly the lack of geo-referenced morbidity data in many countries. This evidentiary gap hinders a fuller understanding of the short-term public health impacts of wildfire smoke and weakens the design of targeted interventions.

A more pressing omission is the absence of data on indigenous and Afro-descendant populations, many of whom reside in or near fire-prone rural territories. Despite being among the most affected by land-use change, deforestation, and climate-exacerbated fire regimes, these communities are virtually invisible in the current evidence base. Their exclusion may stem from limitations in data systems, access to health services, geopolitical marginalisation, or methodological inertia, but regardless of the cause, it constitutes a major equity failure in environmental health research.

Given the demographic and geographic heterogeneity of Latin America, a single summary estimate cannot capture the full public health significance of wildfire smoke exposure. This challenge reinforces the need for high- resolution, locally grounded epidemiological work, ideally integrating air quality modelling, population vulnerability mapping, and engagement with local health authorities and affected communities. In most Latin American countries, morbidity data are aggregated by the location of the health care facility rather than the residence of the patient, complicating efforts to spatially align health outcomes with environmental exposures. This makes it nearly impossible to determine if a spike in emergency visits is occurring in the same neighbourhoods where exposure is most intense. Furthermore, data are often reported at the city or municipality level, without neighbourhood or district-level disaggregation, masking intra-urban disparities. However, there are promising developments. For example, Colombia’s surveillance of severe acute respiratory infections has recently begun to include geographic tags such as zonal planning areas in Bogotá, providing a potential path for future geospatial health analysis.

The findings of this review suggest that the most pressing need is not simply more health effect estimates, but more spatially resolved and representative, equity-informed evidence. Actionable evidence requires studies that extend beyond national averages to capture city-level exposures, neighbourhood-level disparities, and differential susceptibilities across demographic groups. There is potential in exploring morbidity outcomes -such as asthma exacerbations, respiratory infections that require hospitalization, and cardiovascular hospitalisations- as they may better reflect the immediate burden of fire smoke in urban settings than mortality statistics alone.

This review confirms a consistent association between wildfire-related PM_2.5_ and adverse health outcomes in Latin America, particularly increased mortality from respiratory and cardiovascular causes. However, most of the available evidence is limited to national-level analyses or large urban aggregates, often obscuring the spatial and social heterogeneity of exposure and vulnerability. Populations most at risk (such as children, older adults, and historically marginalised ethnic groups) remain under-represented in the existing literature.

Our findings underscore the need for more granular, city-level and submunicipal studies that capture differential health burdens within urban areas, and in rural areas. Given the rising frequency and intensity of fires linked to climate change, especially in periurban and rural-urban interface zones, targeted research is needed to identify where and when wildfire smoke poses the greatest public health threat. Future work should prioritise spatially resolved, equity-oriented morbidity surveillance, leveraging advances in air quality modelling, health informatics, and climate adaptation planning.

## Supplementary archives

**Supplementary table 1.** Newcastle-Ottawa Scale methodological evaluation.

Author (year)	Epidemiological design	Exposure assessment method	NOS (S/C/R)	NOS Total	Quality	Comments
Ye *et al*. ^18^	Time-series	Satellite-derived PM2.5	***/***/***	9	High	Well designed, validated data, controlled for confounding
Chen *et al*. ^10^	Time-series	Satellite-derived PM2.5	***/***/***	9	High	Multi-country, same methodology, good temporal resolution
Ye *et al*. ^31^	Time-series	Satellite-derived PM2.5	***/**/***	8	High	National coverage, good climate control, lower spatial resolution
Gao *et al*. ^32^	Quasiexperimental	Satellite-derived PM2.5	***/**/***	8	High	Robust design (DiD), validated exposure, no individual-level data
Yu *et al*. ^26^	Ecological	Satellite-derived PM2.5	***/**/***	8	High	National coverage, good stratification, limited direct causality
Wu *et al*. ^25^	Meta-regression	Satellite-derived PM2.5	***/**/**	7	High	Well-adjusted for GDP, strong economic analysis, no individual-level data
Butt *et al*. ^29^	Exposure modelling	GEOS-Chem	***/**/*	6	Medium	Emissions well-modelled but without variance or empirical validation
Nawaz and Henze ^28^	Exposure modelling	GEOS-Chem	***/**/*	6	Medium	Broad coverage but low spatial resolution and no validation
Ballesteros-González ^37^	Ecological	WRF-Chem	***/***/**	8	High	Bogotá and region well-represented, but PM_2.5_ underestimated
Nunes *et al*. ^2^	Ecological (crosssectional)	Indirect (FHU, HDI proxies)	**/**/*	5	Medium	Good use of FHU and HDI, but indirect exposure measurement
Cobelo *et al*. ^35^	Ecological	GAM-based modelling	**/**/*	5	Medium	Robust penalization, but no specific wildfire exposure
Gonçalves *et al*. ^33^	Time-series	Satellite-derived PM2.5	**/**/*	7	High	PM_2.5_ well-estimated, acceptable controls, different outcome (COVID)
Lorenz *et al*. ^34^	Ecological (hierarchical)	Satellite-derived PM2.5	**/**/*	5	Medium	GINI and climate included, but unit of analysis is aggregated
Requia *et al*. ^19^	Case-crossover	Satellite-derived PM2.5	***/**/***	8	High	Good confounding control, stratified by quarter

NOS: Newcastle-Ottawa Scale; S/C/R: Selection / Comparability / Outcome or Exposure (depending on study type); DiD: Difference-in-Differences; FHU: Fractional Heat Uptake; HDI: Human Development Index; GAM: Generalised Additive Model; WRF-Chem: Weather Research and Forecasting model coupled with Chemistry; PM_2.5_: Particulate Matter with aerodynamic diameter ≤ 2.5 micrometres; GDP: Gross Domestic Product

Epidemiological designs refer to the analytical framework used to assess health outcomes, while exposure assessment methods describe how wildfire-related PM_2.5_ was estimated. Newcastle-Ottawa scale scores reflect methodological quality based, with higher scores indicating stronger study design. Studies using indirect or unvalidated exposure metrics were rated lower.
